# A101 TRENDS IN MEDICAID SPENDING FOR HEPATITIS C TREATMENT FROM 2012-2021

**DOI:** 10.1093/jcag/gwad061.101

**Published:** 2024-02-14

**Authors:** G Malik, C H Tsai, S E Congly

**Affiliations:** University of Calgary Cumming School of Medicine, Calgary, AB, Canada; Gastroenterology and Hepatology, University of Calgary, Calgary, AB, Canada; Gastroenterology and Hepatology, University of Calgary, Calgary, AB, Canada

## Abstract

**Background:**

Hepatitis C virus (HCV) infection is a pervasive disease that reduces quality and quantity of life. Medicaid is the largest health insurance program in the United States that provides coverage to individuals with low income including children, pregnant individuals, and people with disabilities. Due to the impact of HCV on individuals and the world goal for elimination of HCV by 2030, an analysis of healthcare costs attributed to hepatitis C medications is imperative to guide further policy and coverage changes.

**Aims:**

To analyze Medicaid spending and utilization for hepatitis C treatment from 2012 to 2021 and provide a reflection on the impact it has on healthcare costs in the USA.

**Methods:**

The Centers for Medicare & Medicaid Services public database was accessed to obtain Medicaid spending on Hepatitis C medications from 2012-2021. Data extracted included brand and generic drugs to treat HCV, total annual spending, total dosage units prescribed, total number of claims, and average spending per dosage unit and claim. Microsoft Excel was used for data analysis and creation of graphs.

**Results:**

A total of 20 oral medications were included in this study. Total spending on all medications per year increased from $184 million in 2012 and peaked in 2016 at $3.3 billion. In 2021, the highest amount of Medicaid spending was on Mavyret ($658 million) followed by generic sofosbuvir-velpatasvir ($389 million) and Epclusa ($262 million). The total number of claims decreased from 135,542 in 2012 to 121,101 in 2021 with a peak in 2016 at 159,826. During the same time, the average spending per claim increased from 2012 ($6,518) to 2021 ($146,792). In 2021, the highest average spending per claim was seen with Harvoni ($30,941) followed by Sovaldi ($27,247), Vosevi ($23,877), and Epclusa ($22,917). In the same year, Mavyret had the greatest number of claims at 51,500 followed by generic sofosbuvir-velpatasvir (50,115), Epclusa (11,437), and Vosevi (2,177).

**Conclusions:**

Despite the number of claims declining from 2012 to 2021, the average spending has increased due to the significant cost of HCV medications on Medicaid spending. The highest spending was noted to be with originator medications while generic formulations had the highest number of claims. Understanding the trends in spending on HCV medications can help guide insurance coverage changes for those depending on Medicaid. Lastly, shifting policies towards increased use of generic medications can help with increasing access and reducing costs to the population.

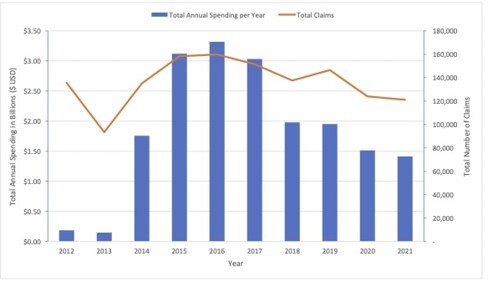

Figure 1. Total annual spending per year in billions ($ USD, blue bars) and total number of claims (orange line) per year from 2012 to 2021.

**Funding Agencies:**

None

